# *Nanos3* knockout pigs to model transplantation and reconstruction of the germline

**DOI:** 10.1111/cpr.13463

**Published:** 2023-04-24

**Authors:** Jing Wang, Jilong Ren, Qingwei Wang, Chongyang Li, Zhiqiang Han, Tianzhi Chen, Ke Sun, Guihai Feng, Ying Zhang, Jianyong Han, Qi Zhou, Wei Li, Dawei Yu, Tang Hai

**Affiliations:** ^1^ Savaid Medical School University of Chinese Academy of Sciences Beijing China; ^2^ State Key Laboratory of Stem Cell and Reproductive Biology, Institute of Zoology Chinese Academy of Sciences Beijing China; ^3^ Institute for Stem Cell and Regenerative Medicine Chinese Academy of Sciences Beijing China; ^4^ University of Chinese Academy of Sciences Beijing China; ^5^ Beijing Farm Animal Research Center, Institute of Zoology Chinese Academy of Sciences Beijing China; ^6^ Institute of Animal Sciences Chinese Academy of Agricultural Sciences Beijing China; ^7^ State Key Laboratory of Agrobiotechnology, College of Biological Sciences China Agricultural University Beijing China; ^8^ Beijing Institute for Stem Cell and Regenerative Medicine Beijing China

Dear Editor,

The anatomy and physiological characteristics of pigs are closer to those of humans than those of other non‐primate animals. Therefore, pigs are considered excellent models for xenotransplantation and disease modelling.^1^ Several pig models of human diseases have been developed using gene editing technology.^2^ However, some of these diseases are too severe to permit the survival and reproduction of pig models.^3^ Therefore, new methods need to be developed to preserve these valuable germlines and genetic resources. Spermatogonial stem cell transplantation (SSCT) is one method to protect the germplasm via germline transfer between male donors and recipients.^4^ Primor germ cells (PGCs) comprise the initial progenitors of the germ cell lineage during development. After a series of complex developmental processes, including epigenetic reprogramming and meiosis, PGCs can develop into sperm cells and oocytes in male and female embryos, respectively.^5^, ^6^, ^7^ Genes such as *Nanos3*, *Tial* and *Dnd1* play important roles in the development of PGCs during their differentiation.^8^, ^9^, ^10^, ^11^ After *Nanos3* is deleted in mice, PGCs undergo apoptosis during migration, resulting in the complete loss of male and female germ cells.^11^ Histological findings revealed no germ cells in the testes or ovaries of *Nanos3* ^mut/mut^ pigs recently created by chimeric embryo injections.^12^ To improve upon chimeric embryo injections, which might yield mosaic pig models, the present study used somatic cell nuclear transfer‐based cloning. The construction of non‐mosaic, germline knockout *Nanos3* ^−/−^ pigs with stable genotypes and phenotypes would be essential for germplasm preservation work.

Here, we constructed a *Nanos3* ^−/−^ pig by somatic cell nuclear transfer (Figure 1A). We initially constructed a *Nanos3* knockout vector (Figure 1B) and cotransfected cultured primary pig embryonic fibroblasts (PEFs) with Cas9‐GFP and an sgRNA for *Nanos3*. We obtained the male cell lines, *Nanos3* ^*−/−*^ #7 and *Nanos3* ^*−/−*^ #1, and a female cell line, *Nanos3* ^*−/−*^ #51 (Figure 1C). Sanger sequencing of the PCR products of these knockout cell lines confirmed the correct genotype (Figure 1D). We then performed somatic cell nuclear transfer and transplanted 4156 reconstructed embryos into 23 surrogates, and 10 were detected using B‐US between 28 and 32 days later. Finally, 18 male and 10 female cloned piglets were born (Figure 1C and Figure S1A). Genotyping of the ear tissues using PCR showed that *Nanos3* was knocked out in all neonatal piglets (Figure 1E). The cloned piglets were raised in a clean, standard environment (Figure S1B). Statistical analysis showed that >70% of the cloned piglets survived for at least 200 days (Figure S1C). These data showed that somatic cell nuclear transfer led to the creation of stable model pigs with complete, constitutive and germline *Nanos3* knockout.

**FIGURE 1:**
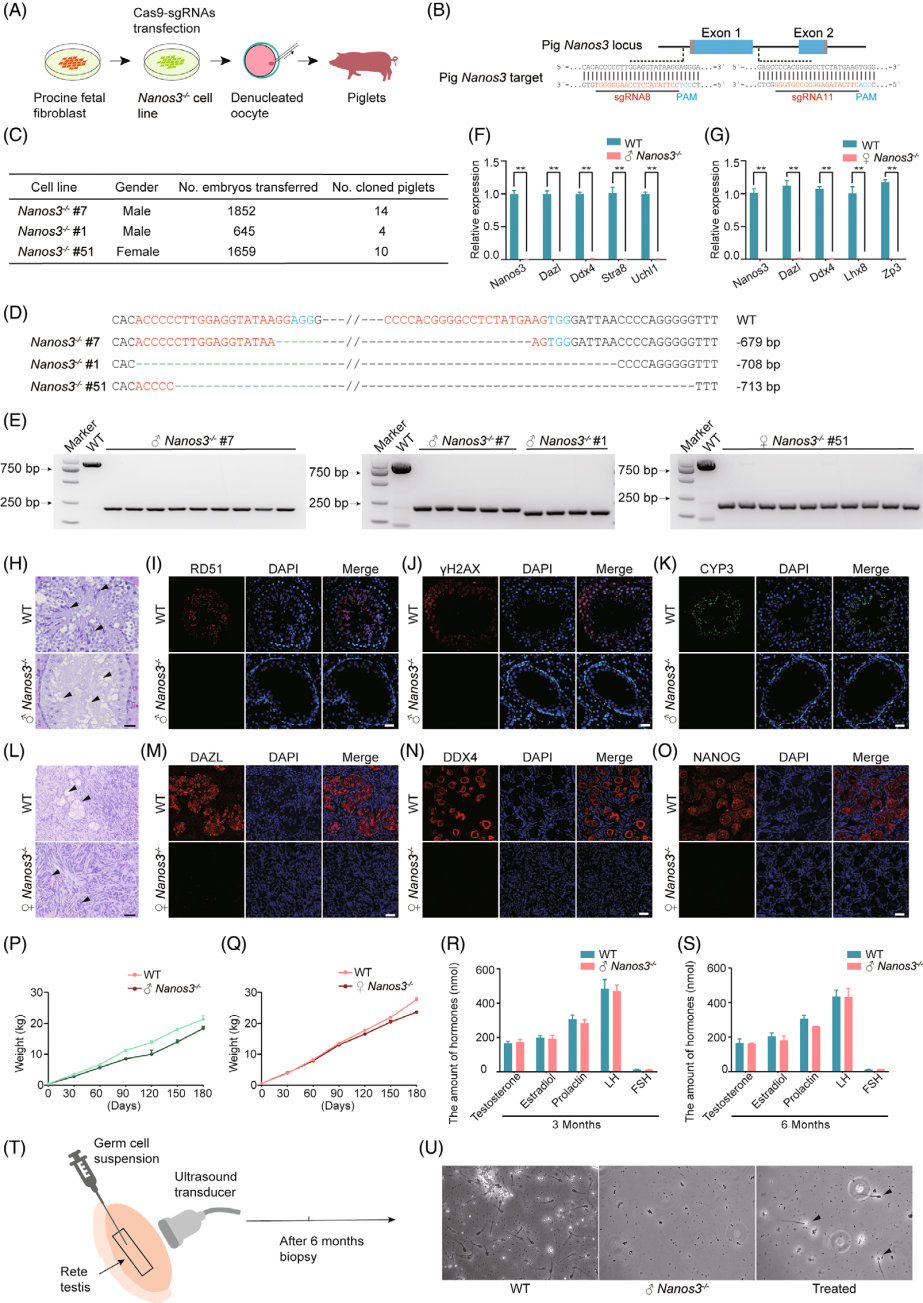
Phenotypes of female and male *Nanos3* ^−/−^ pigs. (A) Experimental procedures involved in the cloning and generation of *Nanos3* ^*−/−*^ pigs. (B) Schematic diagram of CRISPR/Cas9 targeting sites in the *Nanos3* gene. (C) Summary of *Nanos3* ^*−/−*^ cell‐line cloned embryo transfer data required to generate mutant pig models. (D) Sanger sequencing to genotype *Nanos3* ^*−/−*^ cell lines. Deletion (−) sizes are shown on the right of each allele. (E) Genotyping *Nanos3* ^*−/−*^ #7, *Nanos3* ^*−/−*^ #1 and *Nanos3* ^*−/−*^ #51 single cell lines using polymerase chain reaction (PCR). (F) Expression of *Nanos3*, *Dazl*, *Ddx4*, *Stra8*, *Uchl1* in *Nanos3* ^*−/−*^ male pigs (G), and of *Nanos3*, *Dazl*, *Ddx4*, *Lhx8*, *Zp3* in *Nanos3* ^*−/−*^ female pigs. (H) Haematoxylin and eosin (HE)‐stained testicular tissues in 6‐month‐old WT and *Nanos3* ^*−/−*^ pigs. Sperm is undetectable in the testes of *Nanos3* ^*−/−*^ pigs (arrow). (I–K) Immunofluorescence staining for RD51 (red), CYP3 (green) and γH2AX (red) in testes from WT and *Nanos3* ^*−/−*^ pigs. Fluorescent signals are undetectable in *Nanos3* ^*−/−*^ pigs. (L) Ovarian tissues derived from 6‐month‐old female WT and *Nanos3* ^*−/−*^ pigs and stained with HE revealed undetectable follicular structures and oocytes in the latter (arrow). (M–O) Immunofluorescence signals for DAZL (red), DDX4 (red) and NANOG (red) are undetectable in stained ovaries from *Nanos3* ^*−/−*^, compared with WT pigs. (P,Q) Body weight curves for WT and *Nanos3* ^*−/−*^ pigs. (R,S) Hormone levels in 3‐ and 6‐month‐old pigs. (T) Experimental procedures involved in spermatogonial stem cell transplantation (SSCT). (U) Germ cells in WT, *Nanos3* ^*−/−*^, and SSCT pigs.

Next, we analysed the reproductive system of the *Nanos3* ^−/−^ pigs. We stained the testes of *Nanos3* ^*−/−*^ male pigs with haematoxylin and eosin (HE) at the ages of 0 days, 3, and 6 months, and found that spermatogonial stem cells and sperm could not be produced (Figure S1D and Figure 1H). Staining ovarian tissues from *Nanos3* ^*−/−*^ pigs with HE revealed no primary and secondary follicles, or corpus luteum structures at 0 and 3 months (Figure S1E), and no follicle structures and oocytes at 6 months (Figure 1L). We then examined the protein expression of NANOS3 (Figure S1F), DAZL (Figure S1G), and DDX4 (Figure S1H) to confirm the presence of rare germ cells in the testes of *Nanos3* ^*−/−*^ pigs, but no germ cells were detected. We also examined the cell division and differentiation markers (RD51, γH2AX, and CYP3) for spermatogonial stem cells in 3‐month‐old WT testes (Figure 1I–K), but no such cells were detected in *Nanos3* ^*−/−*^ pigs. These findings indicated that *Nanos3* knockout completely disrupted the formation and meiosis of spermatogonia in *Nanos3* ^*−/−*^ pig testes. Moreover, DAZL, DDX4, or NANOG signals were undetectable in newborn and 3‐month‐old female *Nanos3* ^*−/−*^ pigs, indicating the complete absence of female germ cells (Figure S1I–K and Figure 1M–O). We further analysed the expression of genes related to the proliferation, differentiation, and meiosis of spermatogonial stem cells in testes from *Nanos3* ^−/−^ pigs (Figure 1F and Figure S1L). The expression of these genes was significantly decreased in *Nanos3* ^−/−^ compared with wild‐type (WT) pigs. The expression of germ cell‐specific, follicle development‐ and meiosis‐associated genes, as well as the ZP3 gene associated with zona pellucida development was substantially lower in the ovaries of *Nanos3* ^−/−^, compared with WT pigs (Figures 1G and S1M). Body weight (Figure 1P,Q), and levels of testosterone, estradiol, prolactin, luteinizing and follicle‐stimulating hormones (Figure 1R,S) did not significantly differ between WT and *Nanos3* ^−/−^ pigs. Taken together, these data showed that *Nanos3* knockout specifically disrupted the survival, proliferation, and differentiation of germ cells, which did not affect the overall growth and sex hormones of the genetically engineered pigs. Therefore, *Nanos3* ^*−/−*^ pigs can theoretically be used as recipients for SSCT to perpetuate exogenous germlines.

To test this, we injected a suspension of WT spermatogonial stem cells (1 × 10^7^/mL) under ultrasound guidance into the rete testes to verify whether exogenous germ cells can be transplanted and reconstructed in *Nanos3* ^*−/−*^ pig testes (Figure 1T).^13^, ^14^, ^15^ A small but significant amount of mature sperm was detected in the reconstructed testes 6 months later (Figure 1U). This implies that *Nanos3* ^*−/−*^ pig testes can serve as tools for exogenous germ cell reconstruction.

In summary, we found that *Nanos3* knockout specifically and completely disrupted the development of early germ cells in males and females, and that these cells cannot proceed to become functional sperm and oocytes. To the best of our knowledge, this is the first study of *Nanos3* knockout pigs created by somatic cell nuclear transfer. We discovered that constitutive *Nanos3* knockout specifically blocked germ cell development, but did not alter the overall development, growth and survival of *Nanos3* ^*−/−*^ pigs. SSCT for germline preservation could play an important role in changing and maintaining traits among livestock populations, protecting endangered animal species, and combating human infertility in future.^4^ Although Ciccarelli et al. already showed that male *Nanos2* knockout mice, pigs, and goats can support the transplantation of xenogeneic sperm stem cells in vivo and that they can further differentiate into sperm cells,^4^ our present findings also showed an alternative strategy to support the development of SSCT and other technologies for germline reconstruction. While the efficiency and vitality of exogenously transplanted germ cells will need future improvement, we foresee that the addition of *Nanos3* ^*−/−*^ pigs to our toolkit will accelerate such improvements in the future.

## AUTHOR CONTRIBUTIONS

H.T., Y.D., and L.W. conceived and supervised the study. W.J., R.J. and W.Q. designed and performed most of the experiments. W.J., R.J., Y.D., and H.T. analyzed and discussed the data. W.J., and R.J., wrote the manuscript, and L.W., H.T., Y.D., and Z.Y. revised the manuscript. All authors edited the manuscript and approved it for submission.

## CONFLICT OF INTEREST STATEMENT

The authors declare that there are no conflicts of interest.

## Supporting information


**Data S1:** Supporting Information.
