# Supplementary material for: Nanos3 knockout pigs to model transplantation and reconstruction of the germline
Source: Cell Prolif. 2023 Apr 24;56(5):e13463. doi: 10.1111/cpr.13463 (PMC10212706; doi:10.1111/cpr.13463)
Supplement: Supplementary file 1 — Data S1: Supporting Information. [file CPR-56-e13463-s001.docx]

**Supplementary Information**

**Supplementary Figure Legends**

**Supplementary Figures**

**Materials and Methods**

**Supplementary Tables**

**Supplementary Figure Legends**

FIGURE S1

Phenotypic analysis of *Nanos3^-/-^* pig. (A) Embryo transfer of *Nanos3^-/-^* cell lines. (B) *Nanos3^-/-^* pigs derived from *Nanos3^-/-^* #7 cell line. (C) Survival of *Nanos3^-/-^* pigs. Hematoxylin and eosin (HE)-stained 0 and 90-day-old *Nanos3^-/-^* (D) male and (E) female pigs. (F‒H) Immunofluorescence staining of NANOS3, DAZL, DDX4 (red) is undetectable in newborn *Nanos3^-/-^* male pigs. We detected normal supporting cells (GATA4, green), but no germ cells and structurally normal varicose seminiferous tubules in *Nanos3^-/-^* pigs. (I‒K) Immunofluorescence staining for DDX4, DAZL, and NANOG (red) is undetectable in newborn *Nanos3^-/-^* pigs. (L) Expression of *Nanos3*, *Sall4*, *Gfrα1*, *Dmc1*, *Sox17* (associated with the proliferation and differentiation of spermatogonial stem cells) and *Spo11*, *Tnp1*, *Sycp1*, *Prm1*, *Arc* (associated with meiosis) in *Nanos3^-/-^* male pigs. (M) Expression of *Gdf9*, *Gdf9b*, *Foxl12*, *Figla*, *Nobox* (associated with follicular development) and *Cpeb1*, *C-mos*, *Sohlh1* (associated with meiosis) in *Nanos3^-/-^* female pigs.

**Supplementary Figures**


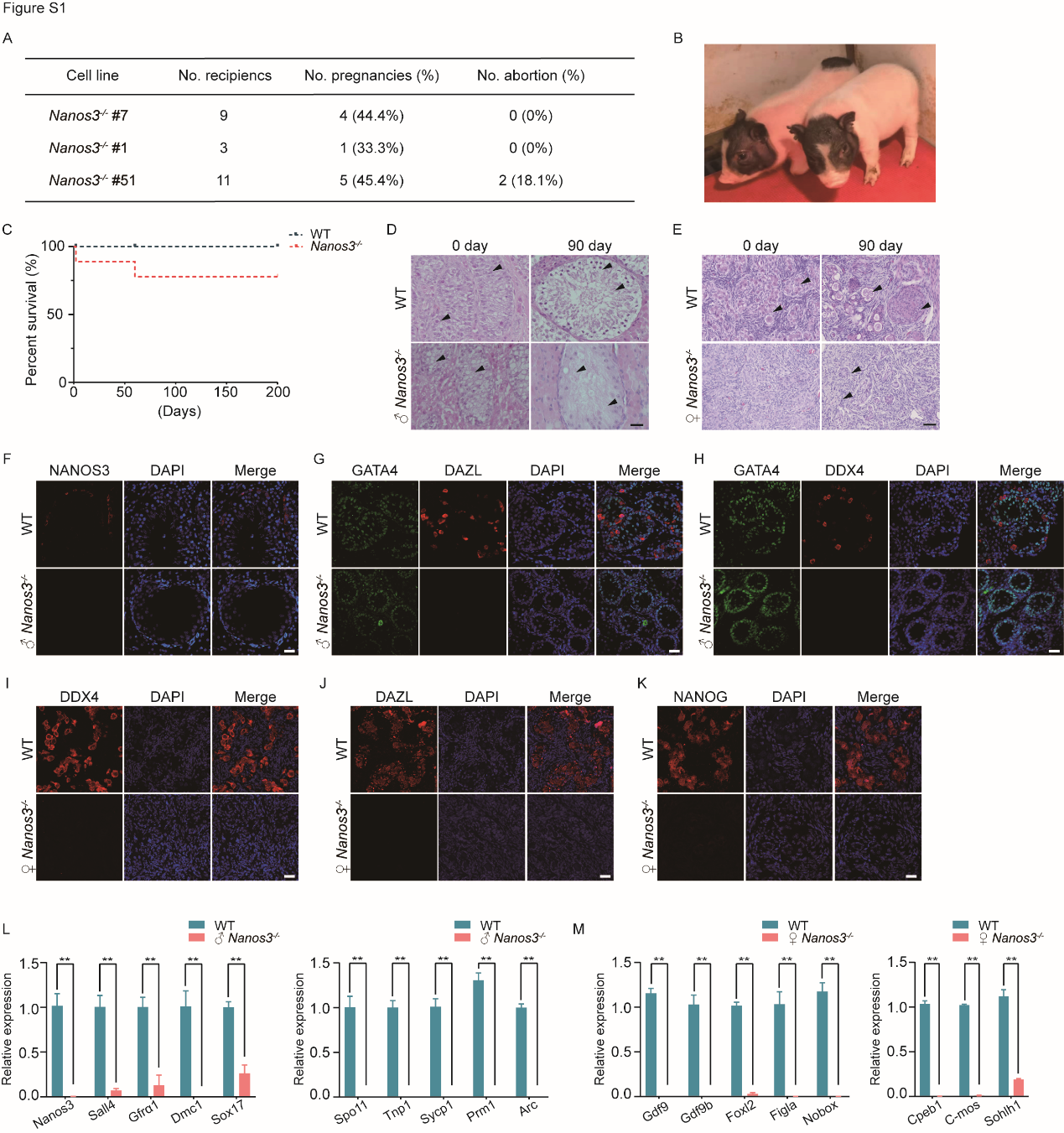


**Materials and Methods**

**Chemicals and animals**

Unless otherwise noted, all chemicals were purchased from Sigma-Aldrich Corp. (St. Louis, MO, USA). All pigs were reared under the Guidelines for the Care and Use of Laboratory Animals Committee of the Institute of Zoology, The Animal Welfare Research Ethics Committee of the Institute of Zoology, Chinese Academy of Sciences, approved the experimental procedures as well as the standards for animal treatment and manipulation (Approval ID: IOZ-IACUC-2021-091).

**Preparation of GFP fibroblasts and cell line screening**

We generated cell lines as described^1^ using the following forward and reverse primers respectively: D-Pig-Nanos3: GGCAGGTGCATTTTTGGAGG and AGCAACAGATCCAGAAGACTGT. The PCR product was 872 bp and the annealing temperature was 60°C. The monoclonal cell line was identified by PCR, and we confirmed that it harbored the correct knockout sequence by Sanger sequencing the product.

**In vitro maturation (IVM), SCNT and embryo transfer**

We conducted IVM, SCNT and embryo transfer as described^2,3^.

**Genotyping**

Genomic DNA extracted from the ear tips of all neonatal piglets using E.Z.N.A.® MicroElute® Genomic DNA Kits (Omega Bio-tek, Inc., Norcross, GA, USA) was amplified by PCR using the *Nanos3*-specific primers described above under the following cycling conditions: 95°C for 5 min, then 35 cycles of 95°C for 30 s, 60°C for 20 s, and 72°C for 30 s, and 72°C for 10 min.

**Reverse transcription PCR**

We extracted RNA using TRIzol, then synthesized cDNA using HiScript II Reverse Transcriptase (R312-01; Vazyme Biotech Co., Ltd., Nanjing, China). The forward and reverse qPCR primers were respectively: Q-*Nanos3*-F: GAAGAGGAACCTGAGACGAGG and Q-*Nanos3*-R: GCACACGTAGTCTCGAAGAATG. The quantitative PCR cycling conditions were: 95°C for 1 min, followed by 40 cycles of 95°C for 10 s, 60°C for 40 s, 95°C for 1 min, 60°C for 30 s, 95°C for 30 s.

**Pathological examination**

Wild-type and *Nanos3^-/-^* porcine testis/ovary tissues were fixed in 4% paraformaldehyde for > 24 h, embedded in paraffin, sectioned, and stained with HE (Thermo Fisher Scientific Inc., Waltham, MA, USA).

**Immunofluorescence staining**

Tissues were fixed and dehydrated in wax blocks, and cut into 5 μm-thick sections. Antigens were retrieved using citric acid antigen repair solution (pH 7.5), then nonspecific antigen binding in the sections was blocked using 10% goat serum. Thereafter, the sections were incubated overnight at 4°C with primary antibody diluted in PBS. The sections were warmed to room temperature and incubated with secondary antibody for 45 min. The sections were washed and nuclei were stained with DAPI. The sections were sealed using a fluorescence anti-quencher, and images were acquired using with a Zeiss 880 microscope (Carl Zeiss Microscopy GmbH, Jena, Germany). The primary and secondary antibodies used were summarized in Supplementary Table S1.

**Donor SSC preparation and transplantation**

We prepared and transplanted donor SSCs as described^4^. Briefly, SSCs were isolated by enzyme digestion^5^, then single-cell suspensions were cultured overnight in DMEM containing 10% FBS. Nonadherent cells (1 × 10^7^/mL) enriched with spermatogonia were suspended in serum-free media and injected into *Nanos3^-/-^* pig rete testes under ultrasound guidance^6,7^.

**Statistics**

All results are those of at least three biological duplications. Data were statistically analyzed using Prism8 (GraphPad Software Inc., San Diego, CA, USA). Between-group values with p < 0.05 were considered statistically significant.

**Supplementary Tables**

**Table S1.** Summary of Antibodies

| Name | Catalog No.; Supplier | Dilution fold |
| --- | --- | --- |
| Anti-DDX4/MVH | Ab13840; Abcam | 300 |
| Anti-DAZL | Ab34139; Abcam | 300 |
| Anti-Nanog | 500-P236-1000; Peprotech | 500 |
| Anti-GATA4 | Sc-1237; Santa cruz | 50 |
| Anti-gamma H2A.X | ab11174; Abcam | 100 |
| Anti-SCP3/SYCP3 | NBP2-23487; Novus | 100 |
| Anti-Nanos3 | PA5-115615; Invitrogen | 100 |
| Anti-RD51 | ab133534; Abcam | 100 |
| Donkey anti-rabbit IgG 488 | A21206; Invitrogen | 1000 |
| Donkey anti-rabbit IgG 594 | A21207; Invitrogen | 1000 |
| Doneky anti-goat IgG 594 | A11058; Invitrogen | 1000 |

1. Fu R, Fang M, Xu K, et al. Generation of GGTA1-/-beta2M-/-CIITA-/- Pigs Using CRISPR/Cas9 Technology to Alleviate Xenogeneic Immune Reactions. *Transplantation.* 2020;104(8):1566-1573.

2. Fu R, Yu D, Ren J, et al. Domesticated cynomolgus monkey embryonic stem cells allow the generation of neonatal interspecies chimeric pigs. *Protein & cell.* 2020;11(2):97-107.

3. Whitworth KM, Lee K, Benne JA, et al. Use of the CRISPR/Cas9 system to produce genetically engineered pigs from in vitro-derived oocytes and embryos. *Biol Reprod.* 2014;91(3):78.

4. Ciccarelli M, Giassetti MI, Miao D, et al. Donor-derived spermatogenesis following stem cell transplantation in sterile NANOS2 knockout males. *Proc Natl Acad Sci U S A.* 2020;117(39):24195-24204.

5. Oatley MJ, Kaucher AV, Yang QE, Waqas MS, Oatley JM. Conditions for Long-Term Culture of Cattle Undifferentiated Spermatogonia. *Biol Reprod.* 2016;95(1):14.

6. Honaramooz A, Megee SO, Dobrinski I. Germ cell transplantation in pigs. *Biol Reprod.* 2002;66(1):21-28.

7. Honaramooz A, Behboodi E, Megee SO, et al. Fertility and germline transmission of donor haplotype following germ cell transplantation in immunocompetent goats. *Biol Reprod.* 2003;69(4):1260-1264.
